# Altered Expression of Par3, aPKC-λ, and Lgl1 in Hippocampus in Kainic Acid-Induced Status Epilepticus Rat Model

**DOI:** 10.3389/fneur.2021.780042

**Published:** 2021-12-08

**Authors:** Chen Zhang, Fafa Tian, Zheren Tan, Juan Du, Xiaoyan Long

**Affiliations:** ^1^Department of Neurology, Xiangya Hospital, Central South University, Changsha, China; ^2^National Clinical Research Center for Geriatric Disorders, Xiangya Hospital, Central South University, Changsha, China

**Keywords:** temporal lobe epilepsy, atypical protein kinase C, partitioning defective 3, lethal giant larvae 1, mossy fiber sprouting

## Abstract

**Introduction:** Mossy fiber sprouting (MFS) is a frequent histopathological finding in temporal lobe epilepsy (TLE) and is involved in the pathology of TLE. However, molecular signals underlying MFS remain unclear. Partitioning defective 3(Par3), atypical protein kinase C-λ(aPKC-λ), and lethal giant larvae 1(Lgl1) were involved in the neuronal polarity and axon growth. The potential roles of those proteins in MFS and epileptogenesis of TLE were investigated.

**Material and Methods:** The epileptic rat models were established by intracerebroventricular injection of kainic acid (KA). The degree of MFS was measured by using Timm staining, Neuronal loss and the expression aPKC-λ, Par3, and Lgl1 in hippocampus were measured by using immunohistochemistry and western blot analysis.

**Results:** The neuronal loss in CA3 region was observed from 3 days to 8 weeks, while the neuronal loss in the hilar region was observed from 1 to 8 weeks in experimental group. The Timm score in the CA3 region in experimental group was significantly higher than that in the control group from 2 to 8 weeks. Compared with control group, the expressions of Par3 and Lgl1 were upregulated and the expression of aPKC-λ was downregulated in the experimental groups. Positive correlation between the Par3 expression and Timm scores, and the negative correlation between the aPKC-λ expression and Timm scores in CA3 region were discovered in experimental group.

**Conclusion:** The findings of the present study indicated that aPKC-λ, Par3, and Lgl1 may be involved in MFS and in the epileptogenesis of temporal lobe epilepsy.

## Introduction

Epilepsy is a devastating neurological disorder. There are estimated 30–40% patients with epilepsy fail to get satisfactory response to appropriate administrations of antiepileptic drugs or other treatments (1). One of the most common types of refractory epilepsy that can be found in adults is the temporal lobe epilepsy (TLE) (2). Therefore, a better understanding for the pathogenesis of TLE is urgently needed. Although exact pathogenic mechanisms of TLE are still unknown, it is generally accepted that the aberrant axonal outgrowth and the synaptic reorganization caused by Mossy Fiber Sprouting (MFS) are the causes of the imbalance between excitatory and inhibitory inputs in hippocampus, which may contribute to the epileptogenesis of TLE (3, 4). Chen et al. discovered that the Repulsive guidance molecule a (RGMa), a protein regulating axonal growth, was significantly decreased in TLE patients, and an overexpression of RGMa in the hippocampus suppresses seizures, MFS and hyperexcitability of hippocampal neurons (5). It was confirmed that a mammalian target of rapamycin (mTOR) pathway was involved in regulating axonal outgrowth (6, 7). Pun et al. selectively removed the mTOR pathway inhibitor phosphatase and tensin homolog (PTEN) gene from an adult-born granule cells, and then a mice without PTEN gene showed spontaneous seizures (8). Based on this finding, LaSarge et al. discovered that PTEN deletion in dentate granule cells resulted in aberrant axonal growth of mossy fiber and enhanced communication between CA3 area and granule cells, thus, resulting to possible promotion of the epileptogenesis of TLE (9). Those findings implicated that axonal growth may be a potential target to attenuate epileptogenesis of TLE.

Numerous studies confirmed that the Partitioning defective 3(Par3) /Partitioning defective 6(Par6)/atypical protein kinase C (aPKC) complex played a determinant role in cell polarity (10–12) and is required for axon–dendrite specification of hippocampal neurons (13–15). The aPKC family has three isoforms: aPKC-ζ, PKM-ζ and aPKC-λ in vertebrate, and Par3 proteins that interacted with aPKC-λ to achieve the function of regulating cell polarity in neuron and in neural progenitors (16–18). In embryonic hippocampal neurons, the aPKC-λ and Par3 complex has localized the presumptive axon (19), which implicated the involvement of aPKC-λ and Par3 in axon specification during the neuronal differentiation. Previous studies had discovered the long-lasting increased of mRNA expression of PKC epsilon in mossy fiber of adult rats, which followed the seizures induced by kainic acid injection (20). Moreover, Gao et al. also discovered that the PAR3 regulates CNTNAP2 spatial localization (21). CNTNAP2 has been considered a prominent disease susceptibility gene associated with epilepsy (22), and seizure was observed in *Cntnap2* knockout rat (23). Those findings implied that Par3 and aPKC-λ have potentiality to be involved in epileptogenesis. However, the functions of aPKC-λ and Par3 in epileptogenesis have not yet been explored.

Lethal giant larvae 1 (Lgl1) was initially known as a tumor suppressor (24) and regulator of polarization processes in variety of cells (25, 26). It has been confirmed that Lgl1 is enriched in developing axons, and upregulation of Lgl1, which has promoted the axonal growth during neuronal morphogenesis (27). Aside from this, Scott et al. found that Lgl1 protein activity is restricted by its phosphorylation by aPKC in the apical neuroblast cortex of flies (28). Nevertheless, this association between Lgl1 and epilepsy has not yet been investigated.

The effects of proteins that were involved in the neuronal polarity are not fully described in epileptogenesis of TLE. By considering the functions of aPKC-λ, Par3, and Lgl1 in neuronal polarity in hippocampal neuron, we also investigated the potential role of those proteins in development of TLE.

## Materials and Methods

### Ethic Statement

All animals were treated humanely. Study design and all animal experimental protocols were done in accordance with the guidelines of the National Institutes of Health, and the study were approved by the Animal Ethics Committee of Central South University (Changsha, China).

### Animals and Model Establishing

Male Sprague–Dawley rats, which are between post-natal days 42 and 56, that weighed between 180 and 220 g, were purchased from the Center for Experimental Animals of Central South University (Changsha, China). The animals were housed under controlled conditions (18-25°C; 50-60% humidity; 12 h light/dark cycle) with food pellets and water available *ad libitum*.

A total of 120 rats were randomly divided into model group (*n* = 65) and control group (*n* = 55) and underwent a surgery for intracerebroventricular injection. Rats were anesthetized with ketamine/xylazine (100/10 mg/kg, I.P.). Rats were placed in a stereotaxic apparatus, and a guide cannula was placed into the lateral ventricle (AP = −0.8, ML = 1.4, DV = 3.3). Kainic acid dissolved in normal saline (0.5 μg/μl) was injected intracerebroventricularly (5 μl/kg, i.c.v.) through a guide cannula by a Hamilton microsyringe at an infusion rate of 0.2 μl/min. The injection usually started 1 min after microsyringe insertion, then cannula will be slowly pulled out of the brain 5 min after the drug injection. Rats in the control group were injected intracerebroventricularly with an equal dose of saline.

The KA-treated rats were observed for the occurrence of seizures immediately following the surgery, and the seizure severity was measured using Racine's scale (29). Five rats died in KA-induced SE. KA-treated rats that demonstrated seizure which reached IV levels and evolved into SE were included in the experimental group (*n* = 60). All saline-treated rats survived after surgery. All rats from experimental and control groups (*n* = 60, and 55, respectively) were sacrificed at five-time points after surgery (3 days and 1, 2, 4, 8 weeks post-surgery) for Timm staining, immunohistochemistry, and western blot analysis.

### Tissue Processing

At different time points, rats were deeply anesthetized by intraperitoneal injection of ketamine/xylazine (100/10 mg/kg). Rats were perfused intracardially with 300 ml saline for immunohistochemistry or with 200 ml 0.4% sodium sulfide in 0.1 M phosphate buffer (pH 7.3) for Timm staining, then followed by application of 4% paraformaldehyde for perfusion fixation. The brains were removed, were fixed in 4% paraformaldehyde for 24 h, then underwent dehydration using 30% sucrose, then finally were cut into 20 μm coronal sections (frozen cryosections). For western blotting, Hippocampus was separated and was stored at −80°C.

### Timm's Staining

In a darkroom, corresponding sections were stained for 90 min in a specific solution which was composed of 100 ml 50% gum arabic, 5 ml citrate buffer (27.2% citric acid and 31% sodium citrate), 15 ml 5.6% hydroquinone, and 0.5 ml 17% silver nitrate. After washing in water, sections were routinely dehydrated, cleaned and mounted with gum. Finally, the corresponding Timm's score for the CA3 region in the hippocampus was analyzed based on the published criteria (30).

### Immunohistochemistry

The tissue sections were incubated at room temperature with 3% H_2_O_2_ solution for 20 min to block the endogenous peroxidase activity. After being washed three times with PBS, the sections were pre-incubated in PBS containing 0.1%Triton X-100 and 5% normal goat-serum for 30 min, then incubated again overnight with the following primary antibodies at 4°C: rabbit anti-aPKC-λ (1:50, Santa cruz), rabbit anti-PAR3(1:300, Abcam), rabbit anti-LGL1(1:40, Santa Cruz), mouse anti- NeuN(1:1,500, Chemicon). After being washed by PBS, the sections were re-incubated with biotin-labeled anti-rabbit or anti-mouse immunoglobulin (Ig) G secondary antibodies which were raised in a goat (1:200, Cwbiotech) at room temperature for 2 h. Afterwards, these were washed in PBS and incubated in avidin-biotin-horseradish peroxidase complex (1:200, Vector laboratories) at room temperature for another 2 h. Finally, the sections were processed with 3,3′-diaminobenzidine tetrahydrochloride solution (Zsbio) for visualization. Following this, the slides were routinely washed, dehydrated, and mounted. Mean optical density (OD) was calculated by Image pro plus 6. The total number of NeuN(+) cells in CA3 and hilar region of five sections, which were randomly selected from every rat, were calculated at 40× magnification of the microscope.

### Western Blotting

Proteins were extracted from hippocampal tissues using RIPA lysate buffer (Beyotime), and then concentrations of proteins were measured using bicinchoninic acid (BCA) Protein Assay Kit (Beyotime). Proteins were separated by using sodium dodecyl sulfate polyacrylamide gel electrophoresis (SDS-PAGE) and then transferred to nitrocellulose membranes. Membranes. which had been washed by using Tris Buffered Saline Tween (TBST) and had been blocked with 5% skimmed milk in TBST (room temperature, 2 h), were incubated at 4°C overnight with primary antibodies. The primary antibodies included rabbit anti-aPKC-λ (1:1,000, Santa Cruz), rabbit anti-PAR3 (1:1,000, Abcam), rabbit anti-LGL1 (1:100, Santa Cruz), and rabbit anti-GAPDH (1:10,000, Proteintech). Unbound antibodies were washed by TBST, then the membranes were subsequently incubated with HRP-labeled goat antirabbit IgG secondary antibodies (1:200, Beyotime) for 1 h. The immunoreactive bands were visualized by using chemiluminescence (ECL) kit (Beyotime), and the optical density (OD) of these bands were quantified by using the Image J software.

### Statistical Analysis

All values were expressed as means ± standard deviations. All statistical analyses were performed using SPSS 21.0 for Windows (SPSS Inc., Chicago, IL, USA). Comparisons between two groups were performed by using two-sample *t* test (parametric) and Mann Whitney *U* test (non-parametric). Multiple comparisons were performed by using one-way analysis of variance and had been followed, respectively, by three more tests; the Least Significant Difference *post-hoc* test (parametric), the Kruskal-Wallis test, and the Bonferroni Procedure (non-parametric). The correlation of MFS severity with expression levels of aPKC-λ, Par3, and Lgl1 were investigated by using Spearman rank correlation coefficient. All tests were two-sided and statistical significance was determined at *p* < 0.05.

## Results

### KA-Induced SE Lead to Neuronal Loss and MFS in Hippocampus

**Figure 1:**
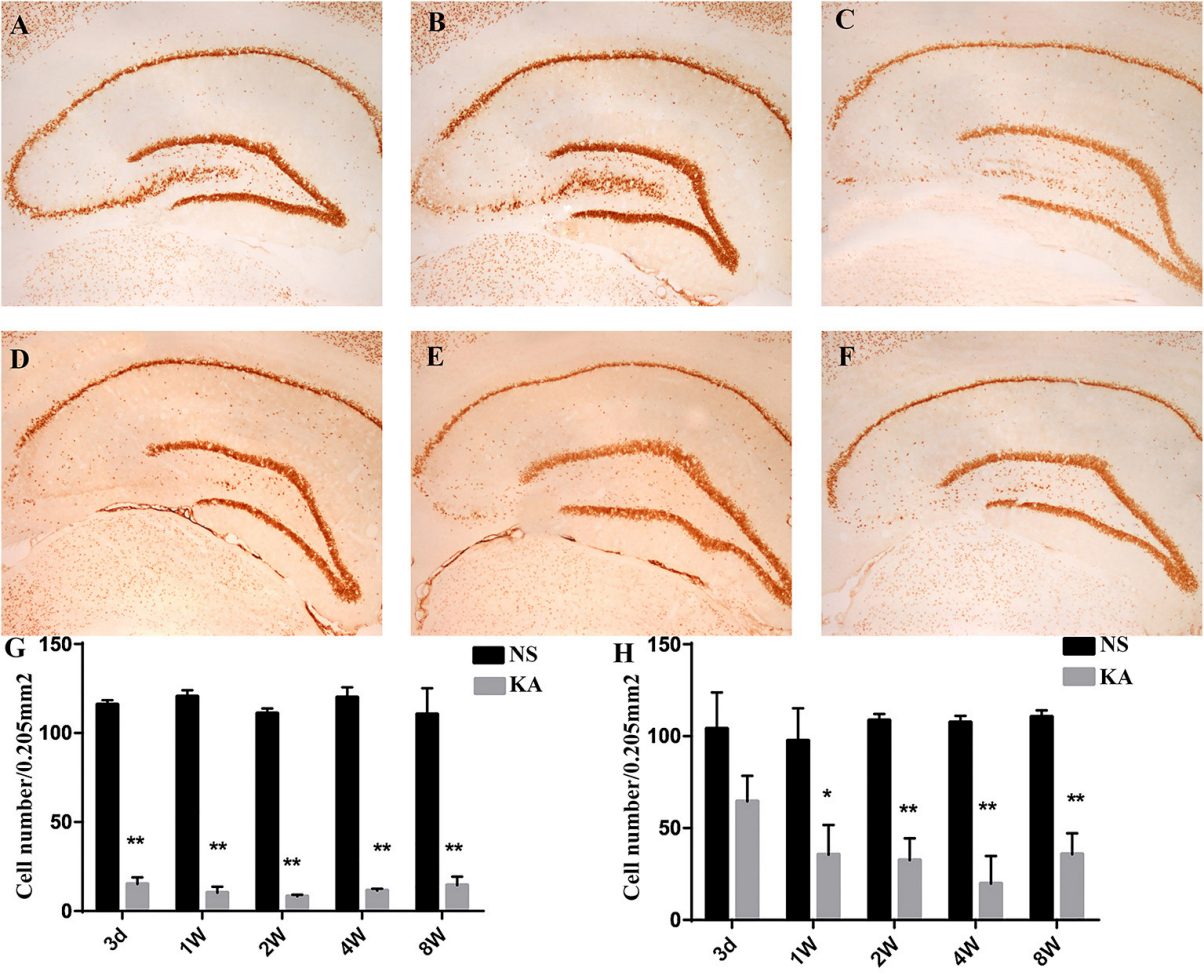
NeuN staining in hippocampus in the control and experimental groups. There is no obvious neuronal loss in control group **(A)**; NeuN staining at five different time points after injection of kainic acid in experimental group: 3 d **(B)**, 1 w **(C)**, 2 w **(D)**, 4 w **(E)**, 8 w **(F)**. The number of NeuN-positive cells measured using NeuN immunohistochemistry in CA3 **(G)** and hilar regions **(H)**. The data are expressed as the mean ± SD. *P < 0.05, compared with the control; and **P < 0.01, compared with the control; KA, kainic acid; NS, normal saline.

The number of NeuN-positive cells, which was measured by using NeuN immunohistochemistry in CA3 and hilar regions, was counted to investigate the neuronal death in hippocampus (Figure 1). Compared with control group, the number of NeuN-positive cells in CA3 region decreased significantly from 3 days to 8 weeks (*p* < 0.001 for all, Figure 1G), and the number of NeuN-positive cells in hilar region decreased significantly from 1 to 8 weeks in experimental group (*p* < 0.01 for all, Figure 1H).

**Figure 2:**
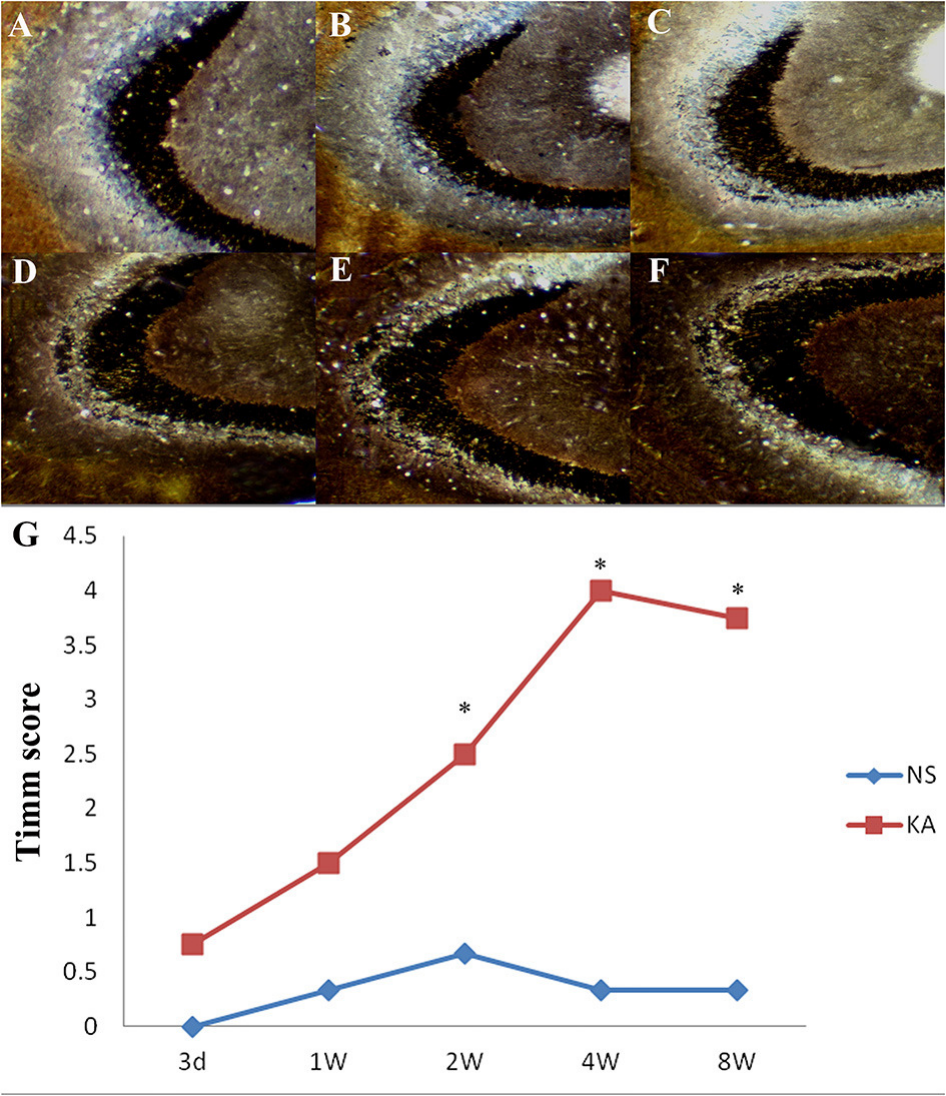
Timm staining in the CA3 region in the control and experimental groups. There is no obvious MFS in control group **(A)**; Timm staining at 5 different time points after injection of kainic acid in experimental group: 3 d **(B)**, 1 w **(C)**, 2 w **(D)**, 4 w **(E)**, 8 w **(F)**. **(G)** Time-dependent changes of Timm scores in the CA3 region in the experimental (*n* = 4 at each time point) and control group (*n* = 3 at each time point). *P < 0.05, compared with the control; KA, kainic acid; NS, normal saline.

Timm staining was used to evaluate the aberrant mossy fiber reorganization in CA3 region (Figure 2), which was graded by Timm scores (a scale of 0–5). In control group, extremely slight MFS appeared in CA3 region from 1 to 8 weeks, and there was no significant difference in Timm scores between all time points (*P* > 0.05). Compared with control group, Timm scores increased significantly from 2 to 8 weeks in experimental group (*P* < 0.05 for all, Figure 2G).

### Expression of aPKC-λ, Par3, and Lgl1 in Hippocampus

There is no significant difference among the all time points with respect to the expression of aPKC-λ, Par3 and Lgl1 in CA3, and hilar region in control group.

**Figure 3:**
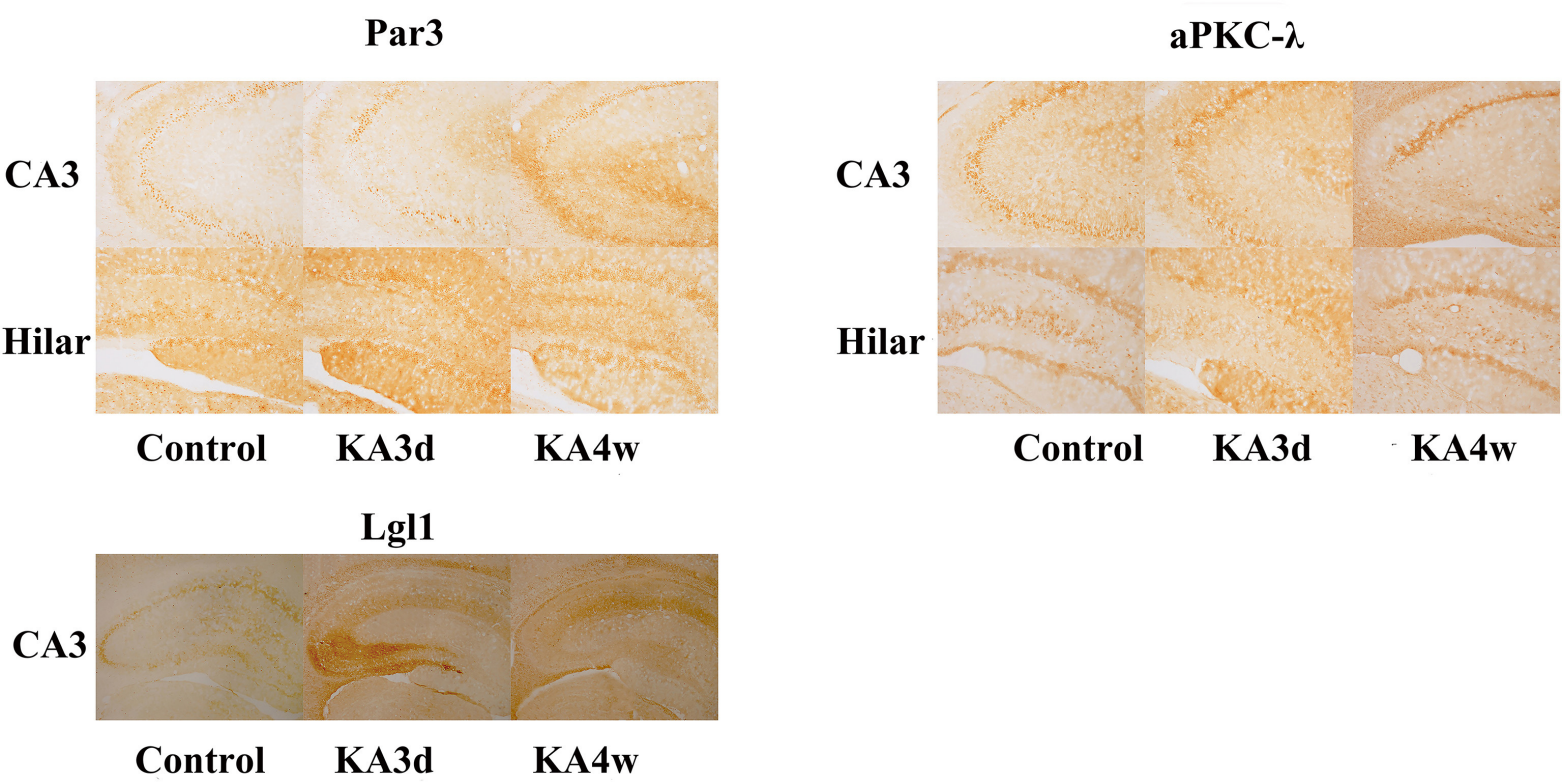
Expression of Par3, aPKC-λ and Lgl1 in the CA3 and hilar region by immunohistochemistry.

Compared with control group, the expression of Par3 in CA3 region decreased significantly in 3 days and increased from 2 weeks to 8 weeks (*p* < 0.05 for all, Figures 3, **5A**), while the expression of Par3 in hilar region decreased significantly in 1 week and increased from 2 weeks to 8 weeks in experimental group (*p* < 0.01 for all, Figures 3, **5B**).

Compared with control group, the expression of aPKC-λ in CA3 region decreased significantly at all time points (*p* < 0.01 for all, Figures 3, **5C**), and the expression of aPKC-λ in hilar region decreased significantly from 2 weeks to 8 weeks in experimental group (*p* < 0.01 for all, Figures 3, **5D**).

The expression of Lgl1 was mainly observed in the cytoplasm in the CA3 region in control group, but the expression of Lgl1 mainly appeared in the axon terminal at CA3 region from experimental group. Compared with control group, the expression of LGL1 in CA3 region increased significantly from 3 days to 4 weeks in experimental group (*p* < 0.05 for all, Figures 3, **5E**).

**Figure 4:**
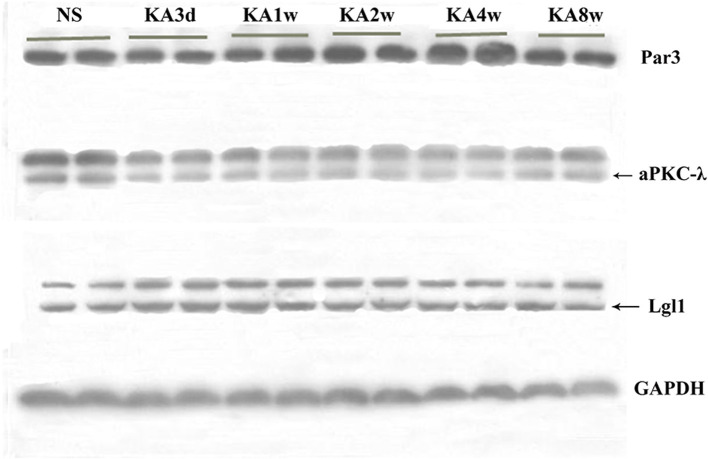
Expression of Par3, aPKC-λ and Lgl1 in hippocampus between experimental and control group by western blot.

**Figure 5:**
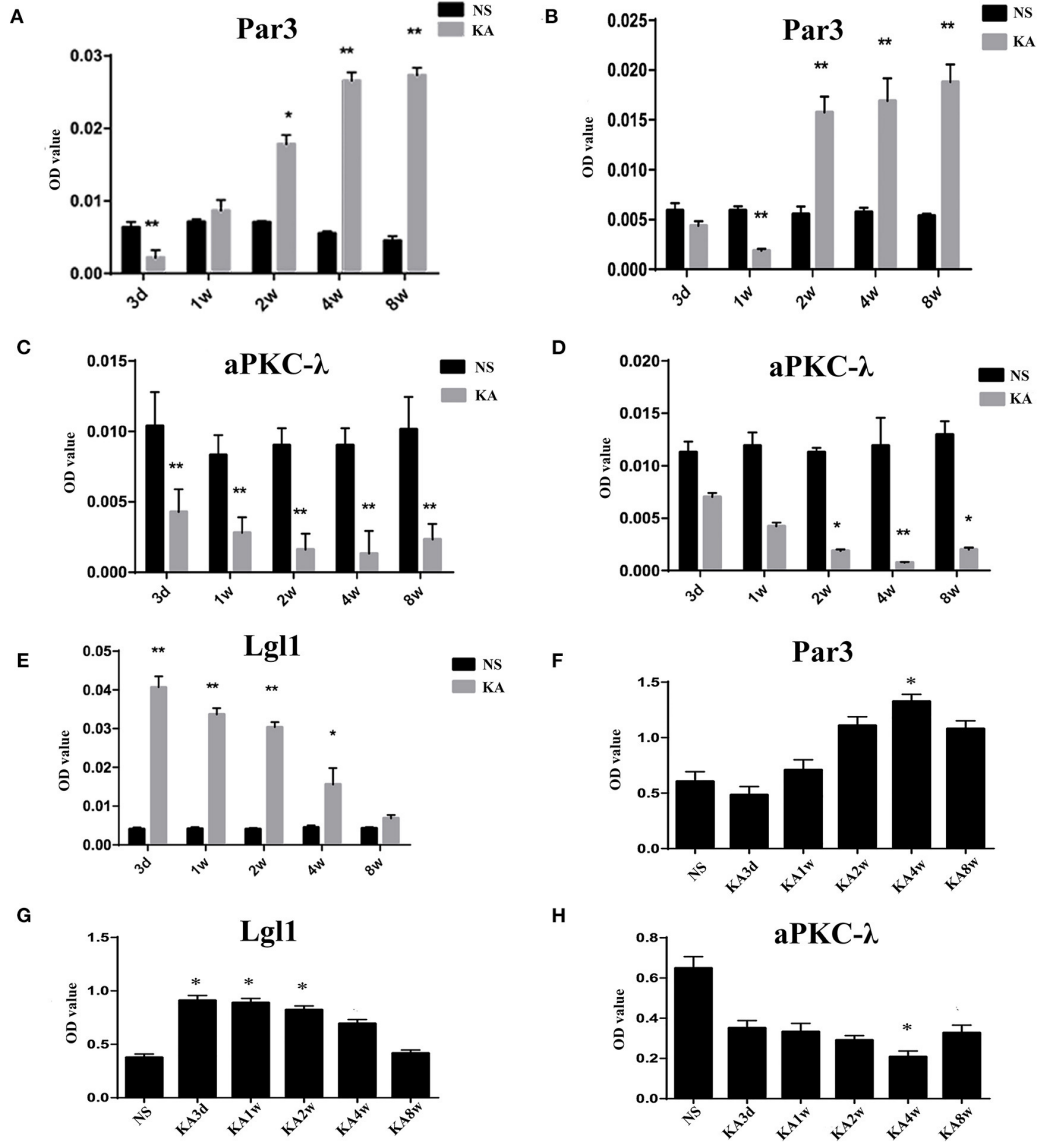
Comparison of Par3, aPKC-λ and Lgl1 in CA3 region **(A,C,E)** and hilar region **(B,D)** between experimental and control group. **(F–H)** Comparison of Par3, aPKC-λ and Lgl1 in hippocampus between experimental and control group.*P < 0.05, compared with the control; and **P < 0.01, compared with the control; KA, kainic acid; NS, normal saline.

The expression of Par3, aPKC-λ, and Lgl1 in hippocampus have been measured by western blot analysis, and multiple comparisons of western blot results were performed (Figures 4, 5). In experimental group, the expression of Par3 in hippocampus increased at 4 weeks (*p* < 0.05, Figure 5F); while the expression of Lgl1 in hippocampus increased significantly from 3 days to 2 weeks (*p* < 0.05 for all, Figure 5G), lastly, the expression of aPKC-λ in hippocampus decreased at 4 weeks (*p* < 0.05, Figure 5H).

**Figure 6:**
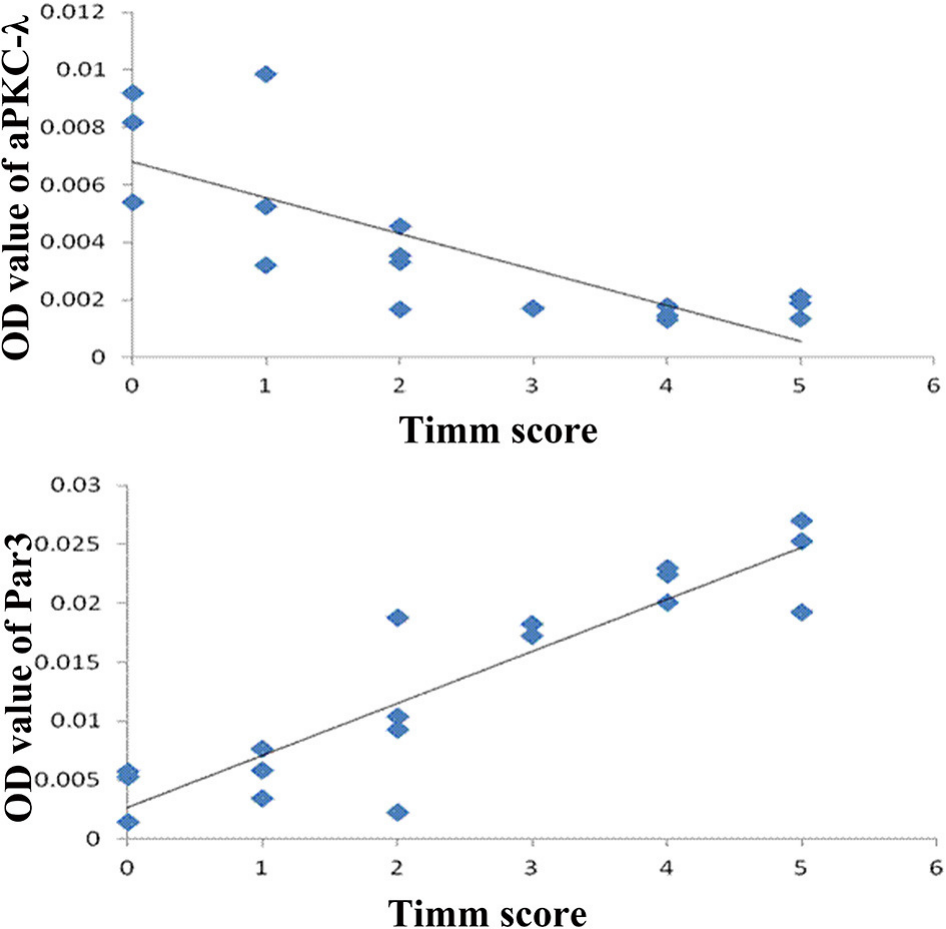
Correlation between Timm score and expression of Par3 and aPKC-λ.

In addition, there is a positive correlation between the expression of Par3 and Timm scores (*r* = 0.903, *P* < 0.01, Figure 6) and a negative correlation between the expression of aPKC-λ and Timm scores (*r* = −0.785, *P* < 0.01, Figure 6) in CA3 region in the experimental group. No correlation between the expression of Lgl1 and Timm scores (*r* = −0.405, *P* > 0.05) was observed in experimental group.

## Discussion

MFS, a frequent histopathological finding in TLE (31), has preceded the appearance of spontaneous seizures in PTZ kindling rat model of epilepsy (32). It has been found that severity of MFS is associated with susceptibility of spontaneous seizures (33, 34). Furthermore, it was confirmed that mTOR pathway was involved in regulating axonal outgrowth (7). In the study conducted by Pun et al., the mice presented MFS and a spontaneous recurrent seizure by deleting PTEN gene that regulates the mTOR pathway (8). All those studies supported the hypothesis that MFS was involved in the mechanism of epileptogenesis of TLE (35–37). Moreover, several studies had discovered that severity of MFS and spontaneous seizures could be relieved by regulating the expression of specific proteins involved in axonal growth (5, 38, 39), which means that further studies are required to explore the role of other proteins that are regulating axonal growth in the mechanism of epileptogenesis of TLE.

Numerous studies confirmed that only two kinds of protein, kinase C (aPKC) isoforms (aPKC-λ and PKM-ζ), were expressed in rat hippocampal neurons, and aPKC-λ-Par3 complex promotes the axon specification, while PKM-ζ competes with aPKC-λ for binding to Par3 and PKMζ-PAR3 complex inhibits axon formation (19, 40), and the upregulating of aPKC-λ or the silencing of PKM-ζ has caused supernumerary axon growth in hippocampal neurons (19). On the contrary, Buchser et al. discovered that the overexpression of aPKC-λ has inhibited neuronal growth and axon formation (41). However, Yamanaka et al. observed that Neuronal deletion of aPKC-λ does not affect distribution of neural structures including dendrites, axons and synapses in mouse brain cortex (42). Those findings indicated that the function of aPKC-λ in axon formation and its growth in neurons are controversial. In the present study, the decrease in aPKC-λ expression at hippocampus and negative correlation between aPKC-λ expression, and the severity of MFS were observed in experimental group. Those findings imply that the condition-dependent effect of aPKC-λ in morphology of axon and aPKC-λ may suppress axonal formation and growth in hippocampus under certain pathological condition (such as epileptogenesis). Besides, effects of aPKC-λ in MFS and epileptogenesis of TLE are still open to debate and further research.

In the present study, Par3 and Lgl 1 increased during epileptogenesis with presence of MFS. Par3 is established as an interaction partner of full-length aPKC isoforms and is involved in neuronal polarity and axon-dendrite differentiation (10–12). It has been demonstrated that the increase in axonal concentration of Par3 could be induced by nerve growth factor or netrin-1 stimulation, which is known to promote axonal growth (43), and the Par3 complex is localized at the presumptive axon in embryonic hippocampal neurons (19). Those findings indicated Par3 may display facilitative effort on MFS and may aggravate epileptogenesis. Moreover, Par3 may indirectly contribute to epileptogenesis through CNTNAP2, which has been considered a prominent disease susceptibility gene associated with epilepsy (21, 22).

Lgl1 is well-known to be a cytoskeletal protein and regulates establishment of polarity in many cell types. It has been confirmed that Lgl1 is enriched in developing axons, and its upregulation promoted the axonal growth (27), which is consistent with findings in present study.

In the present study, the expression of Par 3 in CA3 decreased at 3 days and increased from 2 weeks, then peaked at 8 weeks following KA administration. Conversely, the expression of Lgl1 in CA3 peaked at 3days and declined from 1 week. It reached the lowest level at 8 weeks following KA administration. Those opposite observations may result from antagonism between Par3 and Lgl1 in a cell polarity. Previous studies have confirmed that Par3 and Lgl1/2 all binded directly to Par6 (28, 44–47). However, the binding of Par3 and Lgl to Par6 appeared to be mutually exclusive in establishing and maintaining cell polarization (45–47). Moreover, Wang et al. found that Par3 and Lgl antagonized each other in modulating myosin II activation during cell–cell contact formation (48). Our finding indicated that increase in Par3 and Lgl1 at different phase of epileptogenesis may promote aberrant axonal growth to form excitatory neural connections and aggravate epileptogenesis.

Numerous studies confirmed Par 3 (46) and Lgl (45, 49) could be phosphorylated by aPKC in many cell types. Bailey et al. found that phosphorylation by aPKC prevented lgl from the apical cell cortex in S2 cell system (49). Yamanaka et al. found that activation of aPKC in the Par6 complex resulted in the phosphorylation of Lgl and its dissociation from the cortex in epithelial cell (50). In addition, it has been proposed that the release of Par3 from the Par6/aPKC complex was a result from aPKC-mediated phosphorylation on Par3 via conserved region 3 (51–53). Those findings indicated that the subcellular localizations of lgl and Par3 were regulated by phosphorylation by aPKC, which may have been a possible reason for the expression of aPKC to change in the opposite direction in CA3 and in hilar regions during epileptogenesis compared with lgl and Par3.

In the present study, the neuronal loss in CA3 and hilar region demonstrated by a significant decrease in the number of NeuN-positive cells is observed in the course of epileptogenesis of TLE, which is consistent with previous studies (54, 55). With the neuronal loss in CA3 and hilar region, the loss of connecting targets resulted in the abnormal growth of mossy fiber, and the positive correlation between the degree of neuron loss and extent of MFS has been confirmed in TLE model and patients (56, 57), which indicated that neuronal loss may promote the effect of MFS in epileptogenesis of TLE.

## Conclusions

The findings of this study indicated, for the first time, that aPKC-λ, Par3, and Lgl1 may be involved in MFS and epileptogenesis of TLE. Further studies are required to explore the effects of those proteins mediating cell polarity in MFS and epileptogenesis of TLE, which may lead to more interventions that can prevent or relieve the recurrent spontaneous seizures.

## Data Availability Statement

The raw data supporting the conclusions of this article will be made available by the authors, without undue reservation.

## Ethics Statement

The animal study was reviewed and approved by Research Ethics Committee of the Xiangya Hospital.

## Author Contributions

CZ, ZT, and XL: conceptualization. ZT and CZ: methodology. CZ, ZT, and JD: investigation. CZ: formal analysis, resources, and writing—original draft. FT and XL: writing—review and editing and supervision. All authors have read and approved the manuscript and had full access to all the data in the study, took responsibility for the integrity of the data, and the accuracy of the data analysis.

## Funding

This work was supported by Public Health Program of Hunan Provincial Department of Finance, China (Xiangcaishezhi No. 2020-46, Xiangcaiqizhi No. 2015-122, and Xiangcaijiaozhi No. 2010-216).

## Conflict of Interest

The authors declare that the research was conducted in the absence of any commercial or financial relationships that could be construed as a potential conflict of interest.

## Publisher's Note

All claims expressed in this article are solely those of the authors and do not necessarily represent those of their affiliated organizations, or those of the publisher, the editors and the reviewers. Any product that may be evaluated in this article, or claim that may be made by its manufacturer, is not guaranteed or endorsed by the publisher.
